# Role of Melatonin in Ovarian Function

**DOI:** 10.3390/ani14040644

**Published:** 2024-02-17

**Authors:** Giuseppina Basini, Francesca Grasselli

**Affiliations:** Dipartimento di Scienze Medico-Veterinarie, Università degli Studi di Parma, 43126 Parma, Italy; francesca.grasselli@unipr.it

**Keywords:** indolamine, ovary, ovarian follicle, corpus luteum, reproduction

## Abstract

**Simple Summary:**

Melatonin is a hormone known for regulating the sleep–wake cycle and as an indicator of the photoperiod in seasonally reproducing mammals. Its direct role in ovarian function has been described in the several studies reviewed in the present paper.

**Abstract:**

Melatonin is a hormone mainly produced by the pineal gland in the absence of light stimuli. The light, in fact, hits the retina, which sends a signal to the suprachiasmatic nucleus, which inhibits the synthesis of the hormone by the epiphysis. Mostly by interacting with MT1/MT2 membrane receptors, melatonin performs various physiological actions, among which are its regulation of the sleep–wake cycle and its control of the immune system. One of its best known functions is its non-enzymatic antioxidant action, which is independent from binding with receptors and occurs by electron donation. The hormone is also an indicator of the photoperiod in seasonally reproducing mammals, which are divided into long-day and short-day breeders according to the time of year in which they are sexually active and fertile. It is known that melatonin acts at the hypothalamic–pituitary–gonadal axis level in many species. In particular, it inhibits the hypothalamic release of GnRH, with a consequent alteration of FSH and LH levels. The present paper mainly aims to review the ovarian effect of melatonin.

## 1. Overview

### 1.1. Biosynthesis and Secretion

Melatonin was first isolated from the bovine pineal gland in 1958 [1], and in later years from several animal and plant species. Its extensive distribution, especially in primitive bacteria (cyanobacteria and α-proteobacteria), indicates that this substance is an ancient molecule maintained during the evolution of organisms [2]. In mammals, melatonin secretion is mainly regulated by signals from the nervous system, displaying a circadian rhythm. It is produced and secreted by the pineal gland or epiphysis, a small, well-supplied and innervated endocrine organ located at the base of the brain at the posterior end of the third ventricle. The pineal gland acts as a neuroendocrine transducer, since it is the intermediary between the external environment and the endocrine system, transforming incoming nervous impulses into chemical messages. Sympathetic innervation is essential for the regulation of pineal function and it consists of noradrenergic fibers originating from the cervical sympathetic ganglion and terminating in the interstitial space of the pineal gland or in the plasma membrane of pinealocytes [3]. The sympathetic nervous flow up into the pineal gland is in turn regulated by impulses coming from the suprachiasmatic nucleus of the anterior basal hypothalamus, which is directly controlled by the optic nerve. The signal reaches the endocrine structure, which reacts to the stimulus by releasing hormones at a systemic level. Thus, light regulates epiphysis activity by inhibiting the production of melatonin during the day and maximizing the synthesis of this “hormone of the dark” at night. Circadian rhythms are characterized by oscillations of physiological and metabolic parameters over a 24 h period; the circadian system anticipates environmental changes to optimize the organism’s adaptation based on the time of day, determining a coordinated temporal segregation of numerous biochemical processes in order to make them more efficient. The research that led to the discovery of the molecular mechanisms that control circadian rhythms earned Jeffrey C. Hall, Michael Rosbash and Michael W. Young the Nobel Prize in 2017. As for melatonin secretion, the system is made up of circadian clocks, biological oscillators localized in specific areas of the central nervous system. The organization of these clocks is hierarchical: at the apex we find the master clock, located in the suprachiasmatic nucleus of the hypothalamus, controlled mainly by light signals. Below, we find the secondary brain clocks and peripheral clocks, distributed in various organs and tissues, which are more sensitive to metabolic and nutritional signals. The spontaneous cyclic activity of suprachiasmatic nucleus neurons has been demonstrated, supported by an integrated system of transcriptional and post-translational feedback [4]. It is known that the biosynthesis of melatonin does not only occur at the pineal gland level, but also in the retina, skin and gastrointestinal tract [5,6]. The classic biosynthetic pathway of melatonin in pinealocytes starts from the absorption, from the systemic circulation, of L-tryptophan, which is hydroxylated to 5-hydroxytryptophan by the enzyme tryptophan hydroxylase and subsequently decarboxylated to 5-hydroxytryptamine, better known as serotonin. Serotonin, thus produced, is converted into N-acetyl-serotonin (NAS) by the enzyme N-acetyl-transferase (NAT); the reaction’s product is methylated by the enzyme hydroxyindole-O-methyl-transferase (HIOMT), recently renamed as the acetyl complex amine-O-methyltransferase (ASMT), to form melatonin [7]. In rodents, the NAT enzyme reaches 100-times higher concentrations at night than during the day, and this variation leads to lower levels of serotonin in the dark phase, associated with an increase of N-acetylserotonin and melatonin concentrations [8]. Once synthesized, melatonin is not stored in the pineal gland: the gland’s abundant and impressive blood flow allows for its rapid release into the circulation and, subsequently, into other body fluids such as bile, cerebrospinal fluid, saliva and amniotic fluid [9]. Melatonin detected in follicular fluid could derive from blood circulation or as a result of local ovarian production, since NAT and ASMT are highly expressed in the ovaries [10].

### 1.2. Mechanisms of Action

Melatonin displays a broad functional spectrum of action, as it is involved in various different biological processes. The hormone binds to specific receptors, which are divided into two classes [11]. On the one hand, the family of cell membrane receptors coupled to G proteins, namely the MT1 (MTNR1A) and MT2 (MTNR1B) receptors; on the other hand, the family of quinone-reductase enzymes, including the protein known as MT3 (MTNR1C), or quinone-reductase type 2, which has recently been identified as a cytosolic hormone receptor [12] involved in cell protection against oxidative stress. These receptors were initially identified in the cells of the suprachiasmatic nucleus of the anterior hypothalamus and only later was their expression also found in other organs, demonstrating the multiple biological effects of this hormone [13,14,15]. MT1 and MT2 receptors are G protein-coupled 7-strand transmembrane receptors (GPCRs) that adhere to cell surfaces and therefore interact with external signals. Both have molecular weights of about 40 kDa, a 60% mutual sequence identity, and are encoded by genes on distinct chromosomes. Melatonin binding to MT1 occurs at the cellular level, with inhibitory mechanisms on the cyclic AMP signal transduction cascade (c-AMP) and a consequent reduction in protein kinase A (PKA) activity and CREB protein phosphorylation (c-AMP response element-binding protein), a nuclear transcription factor. MT1 receptors can also couple to the stimulation of the PLC-dependent signal transduction cascade and can activate protein kinase C (PKC). The activation of MT2, resulting from its interaction with the ligand, leads to the inhibition of c-AMP and cyclic GMP (c-GMP) production. The subsequent cellular effects are similar to those dependent on MT1, despite the fact that the latter binds to melatonin with a higher affinity. As previously mentioned, binding to GPCRs is only one of the mechanisms by which melatonin displays its function. In fact, due to its lipophilic nature, the hormone can cross physiological barriers and interact not only with cytoplasmic proteins, but also with nuclear proteins. Among these, RORα and RORβ [16] belong to the family of orphan receptors related to the retinoic acid receptor (RAR). Orphan receptors are so named because their structure is similar to that of other known receptors, but their specific ligand is unknown. Although most of melatonin’s actions are mediated by its specific MT1 and MT2 receptors, a considerable variety of the molecule’s functions depend on its ability to act as an antioxidant. During aerobic metabolism, the production of reactive oxygen species (ROS) occurs mostly at the mitochondrial level and is mainly due to the O_2_ purchase of electrons released from the electron transport chain (ETC): it is estimated that 4% of the O_2_ used in this process is converted to ROS. In the case of oxidative stress, i.e., an imbalance of antioxidants and ROS, toxic effects on cells prevail, with a reduction in ATP synthesis and cell cycle factors, resulting in the aging process of the oocyte [17] and early cell death by apoptosis or necrosis [9]. For this reason, primordial life forms such as bacteria and unicellular organisms evolved neutralization systems, and presumably melatonin at first emerged as an antioxidant and free radical scavenger, later maintaining this function throughout the evolution of all organisms [2]. Actually, it is known that melatonin represents a broad-spectrum non-enzymatic endogenous antioxidant [18]: due to its chemical structure and small size it can act at the mitochondrial level, where most of the ROS are generated, thus maintaining mitochondrial function, preventing DNA damage and the peroxidation of lipids and proteins. In many cases its antioxidant power is greater than that of other antioxidants due, firstly, to its molecular structure, which allows for the donation of electrons or hydrogen atoms, and, secondly, to its associated reaction cascade. Indeed, by interacting with ROS, melatonin generates a variety of metabolites which in turn are able to neutralize toxic oxygen derivatives: cyclic melatonins, hydroxylated melatonins, AFMK (N1-acetyl-N2-formyl-5-methoxy kinuramine) and AMK (N1-acetyl-5-methoxy kinuramine). The antioxidant action of melatonin together with that of its metabolites is defined as a “cascade”, resulting in a protective effect against a large number of ROS [19]. In particular, it is known that AFMK reduces lipid peroxidation, oxidative DNA damage and prevents H_2_O_2_-induced neuronal cell injury; AMK is equally a versatile free radical scavenger capable of inactivating ROS and RNS, acting more effectively as a NO scavenger than melatonin and its metabolite AFMK [20]. Since NO [21] and ROS levels [22] are crucial for ovarian function, these effects have to be taken into account when defining melatonin’s role in the ovary.

## 2. The Role of Melatonin in the Hypothalamic Control of Reproduction

**Figure 1:**
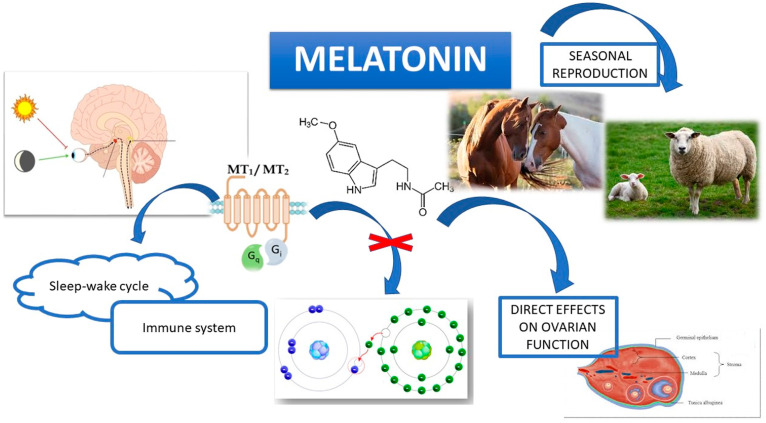
Overview of melatonin’s actions.

Melatonin, due to its cyclical and rhythmic release, is considered a powerful biological clock, a photoperiod indicator and a regulator of the sleep–wake cycle and circadian/yearly rhythms in most mammals [23,24]. Many organisms have adopted a melatonin cycle for this purpose [2]. In fact, unlike unicellular organisms, which directly perceive photoperiod changes and consequently adapt their biological activities, multicellular organisms need a signaling molecule which translates light information into a circadian signal for all cells. Melatonin is widely used in adult humans for the treatment of various sleep disorders, since it displays multiple benefits without the occurrence of tolerance, dependence or side effects at low doses [25,26]. It is commonly utilized for the prevention of jet lag and for the restoration of circadian rhythms. In recent years, several studies have shed light on the possible interaction between melatonin and immunity [27,28]. The pineal gland and melatonin are part of a two-way communication circuit between the neuroendocrine system and the immune system. Many studies have shown that the inhibition of melatonin secretion, achieved by exposure to light at night or the surgical removal of the pineal gland, produces immunosuppression. On the other hand, melatonin treatment at the beginning of the nocturnal period can restore the quality of the body’s antibody response, its resistance to the attack of viruses, parasites and tumor cells and also increase the weight of the thymus [29,30]. The presence of specific binding sites for melatonin on the surface of lymphocyte cells indicates its direct effect on the regulation of the immune system. In fact, T-lymphocytes producing cytokines represent the main mediator of the immunostimulant effects of melatonin. In detail, its actions would include the activation of the production of interleukins-2 and -4 by T lymphocytes, the increase in interleukins (IL-2 and IL-12) production by macrophages and the stimulation of the non-specific immune response, resulting in an increase in the natural killer cells and monocytes numbers in the bone marrow. Those effects are also crucial in reproductive processes, since correct ovarian function strictly depends upon a well-regulated immune response [31,32]. The involvement of melatonin in the management of reproductive rhythms is still not completely known: the molecule acts both at the hypothalamic level, by regulating the secretion of GnRH, as well as at the pituitary level, where the receptors of the molecule are highly expressed, modulating the release of gonadotropins [33,34,35,36]. In fact, some studies suggest that melatonin regulates reproduction by exerting inhibitory effects at the hypothalamic level; on these bases, a receptor-mediated interaction and uptake of the molecule was demonstrated in the rat and hamster hypothalamus [37]. In the rat in particular, melatonin mediates the suppression of GnRH activity in the pituitary and its reduces hypothalamic secretion by 45% through protein kinase A, protein kinase C and MAP-kinase (mitogen-activated protein kinases) [38]. Melatonin’s inhibitory action on gonadotropins’ secretion could represent a control mechanism of the onset of puberty, which would be associated with the ceasing of the suppressive action of high melatonin concentrations on hypothalamic activation; a decrease in melatonin levels below the threshold value provides signals to the hypothalamus that lead to the onset of changes [39]. The melatonin pathway linking SCN and the pituitary is crucial in seasonally reproducing mammals: the photoperiod reading in the SCN is transmitted to the pineal gland via a multisynaptic pathway, resulting in different melatonin secretion profiles, thus converting the light signal into an endocrine rhythm message. Animals with SCN lesions or who are subjected to a surgical removal of their pineal gland are unable to generate photoperiodic responses [40] and their reproductive function is impaired. The variation of daylength provides a “calendar” used by many species to set up reproductive activity during energetically favorable times of the year and with more suitable climatic conditions for the survival of their offspring. The transduction of information on daylength on the neuroendocrine axis is mediated by variations in the nocturnal duration of melatonin secretion [41]. The alterations in indolamine secretion, induced by modifications in daylength, are similar in various species, but result in different effects on the reproductive function of animals with opposite seasonal reproduction periods: in short-day breeders such as sheep and goats [42], an increase in nocturnal melatonin secretion activates the reproductive axis, while it displays an inhibitory effect in long-day breeders [43,44]. In both males and females, melatonin’s role in the control of the reproductive system’s function is not only performed at a central level, but also on the gonads, which represent a target of indoleamine as well as a site of its production. In males [45,46], the molecule acts on the testicular somatic cells, Leydig and Sertoli cells, regulating various physiological activities: in Leydig cells, melatonin acts as a modulator of androgen production, testosterone in particular, while in Sertoli cells it regulates growth, proliferation, energy metabolism, oxidative stress and inflammatory processes, thus playing a fundamental role in steroidogenesis and spermatogenesis, especially in seasonal breeding animals. Therefore, this substance can be administered as an exogenous modulator in cases of testicular pathologies caused by oxidative stress and inflammation [47]. In the female gonads, the plurality of the effects of melatonin reflects the complexity of the physiological and pathological processes taking place at this site, thus making it necessary to further investigate them (Figure 1).

## 3. The Direct Ovarian Role of Melatonin

### 3.1. Follicle

In recent years, the direct involvement of melatonin in ovarian physiology has emerged [48]. Melatonin has been detected in human [36,49] and rat [50] follicular fluid, and its possible role in steroidogenesis at the level of the corpus luteum and granulosa cells has been demonstrated [51,52,53,54]. Melatonin has been found to promote the growth of bovine secondary follicles through its membrane-coupled receptors, while its antagonist luzindole blocks the effects of melatonin on follicle growth and reduces the expression of antioxidant enzymes in cultured follicles [55]. Melatonin acts on secondary follicles by increasing the expression of VEGF and thus promotes follicular angiogenesis, a crucial hallmark in follicular development, by providing new targets for the regulating of follicular development [56]. The substance also plays a role in theca cells, which only express the MT2 receptor, and melatonin treatment has been shown to inhibit androgen biosynthesis [57]. As for theca cells, melatonin has been demonstrated to inhibit the apoptosis and proliferation of in vitro-cultured cells collected from sheep ovaries, thus slowing ovarian atresia and aging. Melatonin uses the PI3K/Akt pathway to mediate the synthesis and secretion of progesterone by theca cells [58]. Abnormal levels of melatonin in theca interna cells might be associated with the development of follicular cysts in sows [59]. The mechanims of protection from the oxidative stress that damages the maturing oocyte and granulosa cells have also been documented [9,60,61]. Xu et al. [62] found that melatonin reduces apoptosis and mitochondrial injury through promoting mitophagy in bovine ovarian granulosa cells, while Zhai et al. [63] suggested a direct involvement of MAP3K8 and the FOS pathway in ovine granulosa cells. The anti-apoptotic action and the increased expression of MT2 in porcine granulosa cells treated with melatonin suggest that the protective effect during follicular atresia results from the binding to membrane receptors and scavenger activity of free radicals [64]. Furthermore, due to its antioxidant action, melatonin can prevent the aging of the porcine post-ovulatory oocyte and supports subsequent embryonic development [17]. This action would suggest a possible use of melatonin to favour the maintenance of an oocyte’s quality in assisted reproduction [33,65,66]. He et al. [64] have documented the presence of the mRNA of MT1 and MT2 receptors in the cumulus oophore of porcine cells as well as a modulatory action of melatonin on porcine granulosa cell function through MT2. A MT2-mediated stimulatory effect on cumulus expansion and subsequent embryonic development has also been shown [67]. In particular, the role of melatonin in humans appears crucial in preventing age-associated germline-soma communication defects, aiding the relay of antioxidant metabolic molecules for the maintenance of oocyte quality from cumulus cells [68]. Melatonin could also represent a promising pharmacological agent for the prevention of the potential toxicity of chemical endocrine disruptors, i.e., exogenous substances that interfere with the production, release and function of the endogenous hormones responsible for maintaining body homeostasis. For example, treatment with Bisphenol A (BPA) has been shown to result in the reduction in the primordial follicle pool, inhibition of follicular growth, impairment of steroidogenesis, and polycystic ovary syndrome [69]. Also, Bisphenol S, analogous to BPA, can disrupt ovarian function since it has been reported to induce a decrease in estradiol production, cell proliferation and the scavenger activity of free radicals in pigs [70]. Wu et al. [69] demonstrated that melatonin appears to specifically mitigate the effects of BPA by increasing estradiol production and granulosa cell proliferation. A positive action of melatonin has also been found with regard to the production of a superoxide anion, resulting from BPA exposure in the oocyte maturation phase. The reduction in mitochondrial apoptosis in pig oocytes treated with the molecule has also been confirmed [71]. Female reproductive health is also threatened by different extremely toxic substances such as Aflatoxins, in particular Aflatoxin B1 (AFB1), one of the most widespread food contaminants affecting public health. Exposure to the substance causes follicular atresia, granulosa cell degeneration, impairment of the oocyte maturation process, increased ROS synthesis and subsequent oxidative stress. The in vitro melatonin treatment of porcine oocytes seems to be the most efficient protective strategy against AFB1-induced toxicity, due to the antioxidant action of indolamine as well as to its ability to inhibit apoptosis and toxin metabolism [72]. Interestingly, Najafi et al. [73] demonstrated that melatonin improves the outcome of ovarian tissue cryopreservation in both the vitrification and slow freezing methods. Moreover, melatonin plays a critical role in reproductive activity and blastocyst implantation in several mammalian species [33]. Implantation is a complex process that requires proper communication between the blastocyst and the uterus through the intervention of cytokines, chemokines, growth factors and steroid hormones, especially estrogen and progesterone. In this regard, melatonin appears to be involved in the regulation of various phases of the process; treatment with the molecule features the regulation of apoptotic mechanisms in embryonic cells at the ICM (Inner Cell Mass) level, the increase in potent adhesion protein expression that favors blastocyst implantation and development and, finally, protection from oxidative stress, which decreases embryo quality and pregnancy success [34,35]. Melatonin has been detected in follicular fluids, with decreased levels occurring in women with polycystic ovary syndrome (PCOS) [49]. Studies have documented the presence of melatonin in porcine follicular fluid, whose physiological concentration decreases proportionally to follicular growth. Levels of 20 pg/mL in the follicular fluid of small follicles and 10 pg/mL in that of large ones have been documented, suggesting melatonin’s involvement in oocyte maturation as well [74]. Therefore, Basini et al. [51] investigated the effects of melatonin in porcine granulosa cells collected from large and small follicles by evaluating their proliferation, steroidogenesis and angiogenesis. The study confirmed a possible involvement of melatonin in the local modulation of the ovarian follicle’s function. In particular, the P4 production in cells collected from large follicles was inhibited by 20 pg/mL melatonin. On this basis, it is possible to assume that the low physiological concentration detected in the fluid of this class of follicles (10 pg/mL) could favour the luteinization process by stimulating progesterone synthesis. Since knowledge about melatonin’s role in the control of the corpus luteum’s function is scarce, and experimental evidence is sometimes conflicting, future studies are needed in humans, as well as in different animal species.

### 3.2. Corpus Luteum

**Figure 2:**
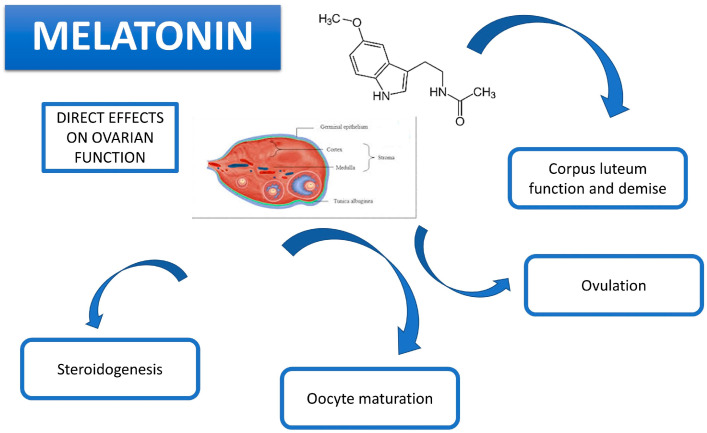
Main effects of melatonin on the ovary.

As previously highlighted, the involvement of melatonin in protection from oxidative stress within the ovary is now known, but this aspect awaits further investigation in the corpus luteum. It has been demonstrated that a melatonergic system is essential for luteal function in mammals [75]. Reactive oxygen species (ROS), which mainly derive from the reactions of normal metabolism, are involved in the process of luteogenesis and luteolysis [76]. In particular, during the transport of cholesterol for progesterone synthesis, as well as in the regression phase, lipid peroxidation results in luteal plasma membrane damage, oxidative stress-related cell apoptosis and an inhibition of luteal function [77,78,79,80]. Melatonin, as a free radical scavenger, protects lutenizing granulosa cells in the ovulatory follicle from ROS and contributes to the process by potentiating the production of progesterone after ovulation, while also preventing premature luteolysis due to oxidative stress in the newly formed corpus luteum [79,80,81]. The higher concentration of indolamine detected in human serum during the luteal phase compared to the follicular phase confirms the hypothesis of a direct effect of the molecule on this process. Scarnici et al. [53] investigated different parameters in the human corpus luteum before and after treatment with melatonin, reporting an increase in progesterone concentration as well as MT2 receptor expression. The potential of this hormone has been also investigated in other species such as the sheep, in which, over the years, important information has been acquired with possible implications for humans. In the ovine corpus luteum, it is possible to find high concentrations of the enzymes involved in melatonin synthesis as well as the expression of both receptors, thus confirming this site as a potential area of hormone synthesis and a target of the hormone [81]. Furthermore, it has been shown that melatonin deficiency causes a reduction in plasmatic antioxidant capacity in sheep and an impairment of the dynamics of follicular and luteal growth, with a reduction in progesterone synthesis [82]. Melatonin has been demonstrated to promote progesterone secretion in sheep luteal cells by regulating autophagy via the AMPK/mTOR pathway [83]. Other authors, on the contrary, have documented no correlation or negative effects of melatonin on P4 synthesis during the growth and luteinization of granulosa cells. Both the MT1 receptor’s mRNA and protein are expressed in equine corpus lutea. Melatonin inhibits the production of progesterone, as well as the expression of P450scc, in equine luteal cells, and the effect is dose-dependent. The inhibitory effect of melatonin is blocked by luzindole, a non-selective melatonin receptor antagonist. The data support the presence of functional melatonin receptors in luteal cells and a regulatory role of melatonin in the endocrine function of the equine CL [84]. In exploring its direct effect on P4 secretion in human granulosa cells subjected to defined culture conditions, a suppression of P4 output and, therefore, a hypothetical regulatory effect on granulosa function in the menstrual cycle seems evident [85]. Similar results emerged in an earlier study on granulosa cells isolated from pig ovaries and treated with melatonin at different concentrations [86]. Presumably, the negative effect on P4 production could be prevalent in the early stages and be due to the inhibition of cAMP, a secondary messenger in the steroidogenesis pathway; in later stages, this effect’s results are overcome by upregulatory and stimulatory effects [87]. The direct action of melatonin on luteal function has also been evaluated in earlier stages and during pregnancy, since the importance of this transient endocrine organ in the first stages of gestation is well known [88]. In heat-stressed cows, melatonin can improve luteal haemodynamics [89]. A local synthesis of melatonin in the luteal cells of pregnant sows has been documented, thus suggesting a paracrine and/or autocrine role of melatonin in luteal function [90]. The expression of hormone receptors in porcine luteal cells during pregnancy and an increase in progesterone levels proportional to the melatonin concentration, and mediated by the up-regulation of P450scc and StAR, has been demonstrated [91]. Moreover, the stimulatory effects of melatonin on GnRH and LH production in the luteal cells of pregnant sows has been reported, suggesting a potential role for melatonin in luteal function through regulating the release and synthesis of GnRH and LH in luteal cells [92]. In the mouse model, it has been documented that the administration of melatonin upregulates the genes involved in the synthesis of pregnenolone, promotes corpus luteum development before gestation, favors embryonic implantation and increases uterine receptivity in the early stages of pregnancy (Figure 2) [93].

## 4. Conclusions

Melatonin plays a fundamental role in rhythmicity and reproductive seasonality in mammals. It is widely known that ovarian function is controlled by the hypothalamic–pituitary–gonadal axis, but a growing body of literature supports the hypothesis of a direct action of melatonin on mammalian ovaries. The effects of indoleamine on the ovary are exerted through the activation of specific G-protein coupled receptors and/or by its unique action as a potent scavenger of free radicals. As depicted in Figure 1, melatonin has been demonstrated to be directly involved in several ovarian physiological mechanisms, such as steroid hormone synthesis, oocyte maturation, ovulation and corpus luteum formation and demise. However, the molecular pathways and machineries underlined by these specific events are mostly unknown. Further studies aiming to better define the roles of indoleamine in ovarian physiology should clarify the direct actions of melatonin at the mechanistic level, thereby helping to fully develop the therapeutic potential of this substance in combating infertility.

## Data Availability

Data sharing is not applicable as no new data were generated or analyzed during this study.
